# Supplementary material for: Post-Translational Modifications of Histone Variants in the Absence and Presence of a Methionine-Depleting Enzyme in Normal and Cancer Cells
Source: Cancers (Basel). 2023 Jan 15;15(2):527. doi: 10.3390/cancers15020527 (PMC9857184; doi:10.3390/cancers15020527)
Supplement: Supplementary file 1 [file cancers-15-00527-s001.zip › cancers-2104543-supplementary.pdf]

Table S1. Effects of MGL50 on PTMs of H1 histone variant in Hs27 and HT-29 cells. Cells were seeded (2.5×10^5 cells/well) into 12-well plates, were treated with 0.03 mg/mL MGL (MGL50) for 48 h and then were collected. Histones were extracted and peptides were analyzed by mass spectrometry. Data are represented as mean and standard error. NT, non-treated; MGL50, concentration of MGL able to reduce cell viability to 50%. Heteroscedastic two-tails t-test are applied and statistical analysis was carried out comparing Hs27 treated vs not treated, HT-29 treated vs not treated, HT-29 non treated vs Hs27 non treated. p-value are reported as -log2. Statistically significant differences are highlighted in green.

	mean				standard error									
	Hs27 cells		HT-29 cells		Hs27 cells		HT-29 cells							
	NT	MGL50	NT	MGL50	NT	MGL50	NT	MGL50	Hs27 MGL50/NT	p-value	HT-29 MGL50/NT	p-value	HT29/Hs27 NT	p-value
H14_25_32 unmod	65.51%	76.43%	62.38%	61.05%	13.62%	7.39%	5.28%	13.86%	0.22	1.19	-0.03	0.12	-0.07	0.31
H14_25_32 K25me1	25.55%	18.21%	31.73%	27.08%	13.95%	7.10%	5.97%	14.57%	-0.49	0.74	-0.23	0.44	0.31	0.63
H14_25_32 K25me2	0.00%	0.00%	0.00%	0.01%	0.00%	0.00%	0.00%	0.01%	0.00	0.00	0.00	0.00	0.00	0.00
H14_25_32 K25me3	0.50%	0.41%	1.62%	1.51%	0.24%	0.14%	0.12%	1.15%	-0.27	0.46	-0.10	0.12	1.69	7.15
H14_25_32 K25ac	2.75%	0.22%	1.89%	1.63%	1.36%	0.12%	0.73%	1.22%	-3.67	2.74	-0.21	0.26	-0.54	0.90
H14_25_32 K31ac	4.94%	3.42%	2.38%	8.04%	1.09%	0.45%	0.71%	3.96%	-0.53	2.39	1.75	2.64	-1.05	3.75
H14_25_32 S26ac	0.74%	1.31%	0.00%	0.68%	0.65%	1.60%	0.00%	0.84%	0.82	0.50	0.00	0.00	0.00	0.00
H14_25_32 S26ph	0.01%	0.00%	0.00%	0.00%	0.02%	0.00%	0.00%	0.00%	0.00	0.00	0.00	0.00	0.00	0.00
H12_33_53 unmod	59.22%	67.57%	60.12%	70.05%	1.19%	2.82%	4.58%	0.44%	0.19	5.11	0.22	3.11	0.02	0.27
H12_33_53 K33me1	40.60%	32.43%	39.72%	29.91%	1.16%	2.82%	4.60%	0.46%	-0.32	5.03	-0.41	3.07	-0.03	0.27
H12_33_53 K33me2	0.02%	0.00%	0.09%	0.04%	0.03%	0.00%	0.05%	0.02%	0.00	0.00	-1.31	1.94	1.89	2.27
H12_33_53 K33me3	0.15%	0.00%	0.05%	0.00%	0.06%	0.00%	0.05%	0.00%	0.00	0.00	0.00	0.00	-1.72	2.69
H12_33_53 K33ac	0.00%	0.00%	0.02%	0.00%	0.00%	0.00%	0.03%	0.00%	0.00	0.00	0.00	0.00	0.00	0.00
H15_36_56 unmod	22.33%	16.11%	22.57%	16.88%	0.29%	4.84%	3.52%	0.55%	-0.47	1.97	-0.42	2.45	0.02	0.09
H15_36_56 K36me2	0.01%	0.00%	0.01%	0.00%	0.01%	0.00%	0.02%	0.00%	0.00	0.00	0.00	0.00	0.24	0.13
H15_36_56 K36me3	38.75%	42.50%	38.63%	41.48%	0.14%	2.17%	1.77%	0.26%	0.13	2.58	0.10	2.43	0.00	0.08
H15_36_56 K36ac	38.38%	41.39%	38.59%	41.44%	0.46%	2.72%	1.82%	0.24%	0.11	1.99	0.10	2.37	0.01	0.16
H15_36_56 S43ac	0.53%	0.00%	0.19%	0.20%	0.50%	0.00%	0.05%	0.05%	0.00	0.00	0.10	0.26	-1.51	1.03
H1_1_35 H12.SETAPAAPAAAPPAEKAPVKKKAAKKAGGTPR	0.90%	0.30%	0.06%	0.26%	0.99%	0.37%	0.07%	0.31%	-1.57	0.92	2.07	1.00	-3.88	1.29
H1_1_35 H14.SETAPAAPAAPAPAEKTPVKKKAR	66.91%	71.50%	74.49%	49.38%	2.46%	11.84%	3.25%	20.63%	0.10	0.59	-0.59	1.87	0.15	3.56
H1_1_35 H15.SETAPAETATPAPVEKSPAKKKATKKAAGAGAAKR	32.19%	28.19%	25.45%	50.37%	1.62%	11.84%	3.18%	20.32%	-0.19	0.47	0.98	1.89	-0.34	3.61
H1_54_81 H11.GGVSLAALKKALAAAGYDVEKNNSR	0.00%	33.87%	0.06%	1.02%	0.00%	20.91%	0.07%	0.68%	0.00	0.00	3.98	2.14	0.00	0.00
H1_54_81 H1v234.SGVSLAALKKALAAAGYDVEKNNSR	78.16%	62.15%	79.64%	68.16%	13.54%	23.41%	6.28%	3.56%	-0.33	0.98	-0.22	3.02	0.03	0.14
H1_54_81 H15.NGLSLAALKKALAAGGYDVEKNNSR	21.84%	3.98%	20.29%	30.82%	13.54%	2.52%	6.27%	2.88%	-2.46	2.41	0.60	2.89	-0.11	0.14

Table S2. Effects of MGL50 on PTMs of H2A histone variant in Hs27 and HT-29 cells. Cells were seeded (2.5×10^5 cells/well) into 12-well plates, were treated with 0.03 mg/mL MGL (MGL50) for 48 h and then were collected. Histones were extracted and peptides were analyzed by mass spectrometry. Data are represented as mean and standard error. NT, non-treated; MGL50, concentration of MGL able to reduce cell viability to 50%. Heteroscedastic two-tails t-test are applied and statistical analysis was carried out comparing Hs27 treated vs not treated, HT-29 treated vs not treated, HT-29 non treated vs Hs27 non treated. p-value are reported as -log2. Statistically significant differences are highlighted in green.

	mean				standard error									
	Hs27 cells		HT-29 cells		Hs27 cells		HT-29 cells							
	NT	MGL50	NT	MGL50	NT	MGL50	NT	MGL50	Hs27 MGL50/NT	p-value	HT-29 MGL50/NT	p-value	HT29/Hs27 NT	p-value
H2A1_36_42 unmod	98.62%	99.65%	99.73%	99.90%	0.91%	0.43%	0.14%	0.12%	0.02	1.84	0.00	1.74	0.02	1.87
H2A1_36_42 K36ac	0.05%	0.00%	0.27%	0.10%	0.06%	0.00%	0.14%	0.12%	0.00	0.00	-1.49	1.74	2.42	2.80
H2A1_36_42 Y39ac	1.33%	0.35%	0.00%	0.00%	0.87%	0.43%	0.00%	0.00%	-1.93	1.83	0.00	0.00	0.00	0.00
H2A3_36_42 unmod	66.67%	0.00%	100.00%	95.91%	40.82%	0.00%	0.00%	5.00%	0.00	0.00	-0.06	0.00	0.58	0.00
H2A3_36_42 Y39ac	0.00%	0.00%	0.00%	4.09%	0.00%	0.00%	0.00%	5.00%	0.00	0.00	0.00	0.00	0.00	0.00
H2AX_36_42 unmod	100.00%	66.67%	98.99%	98.86%	0.00%	40.82%	1.24%	1.40%	-0.58	0.00	0.00	0.10	-0.01	0.00
H2AX_36_42 Y39ac	0.00%	0.00%	1.01%	1.14%	0.00%	0.00%	1.24%	1.40%	0.00	0.00	0.18	0.10	0.00	0.00
H2A1_4_11 unmod	73.43%	85.29%	93.18%	87.47%	2.46%	8.14%	1.37%	5.15%	0.22	2.62	-0.09	1.95	0.34	9.95
H2A1_4_11 K5ac	0.61%	0.23%	1.76%	1.15%	0.74%	0.23%	1.07%	0.72%	-1.40	0.78	-0.62	0.75	1.53	1.55
H2A1_4_11 K9ac	1.66%	1.48%	1.26%	0.86%	0.77%	0.86%	0.79%	0.54%	-0.17	0.23	-0.56	0.66	-0.39	0.55
H2A1_4_11 K5acK9ac	0.01%	0.00%	0.22%	0.26%	0.01%	0.00%	0.05%	0.11%	0.00	0.00	0.22	0.46	4.96	7.29
H2A1_4_11 K9me1	13.59%	6.33%	0.98%	2.30%	4.18%	6.30%	0.80%	2.74%	-1.10	1.71	1.23	0.74	-3.80	5.49
H2A1_4_11 K5me1	10.70%	6.68%	2.60%	7.97%	1.74%	4.14%	0.39%	3.84%	-0.68	1.58	1.62	2.13	-2.04	7.61
H2AJ_4_11 unmod	6.33%	0.90%	19.01%	8.84%	4.15%	0.59%	0.51%	7.12%	-2.81	2.01	-1.10	2.17	1.59	4.00
H2AJ_4_11 K5ac	0.07%	0.21%	0.05%	3.14%	0.03%	0.25%	0.04%	0.72%	1.64	0.86	6.10	4.89	-0.58	0.68
H2AJ_4_11 K9ac	28.53%	22.10%	9.13%	7.59%	20.80%	9.90%	6.53%	8.37%	-0.37	0.42	-0.27	0.21	-1.64	1.57
H2AJ_4_11 K5acK9ac	0.02%	0.00%	0.00%	10.44%	0.03%	0.00%	0.00%	12.68%	0.00	0.00	11.85	1.25	-3.09	1.09
H2AJ_4_11 K9me1	16.46%	0.22%	13.86%	1.13%	6.89%	0.18%	8.43%	0.95%	-6.24	3.29	-3.61	2.29	-0.25	0.35
H2AJ_4_11 K5me1	48.59%	76.57%	57.95%	68.86%	23.72%	9.54%	1.79%	18.41%	0.66	1.99	0.25	0.88	0.25	0.56
H2AX_4_11 unmod	5.90%	3.88%	6.30%	9.17%	0.69%	0.19%	1.39%	4.79%	-0.60	5.29	0.54	0.94	0.09	0.37
H2AX_4_11 K5ac	0.27%	0.00%	0.17%	0.10%	0.16%	0.00%	0.03%	0.10%	0.00	0.00	-0.83	1.19	-0.66	1.01
H2AX_4_11 K9ac	0.12%	0.02%	0.08%	0.05%	0.08%	0.03%	0.02%	0.04%	-2.41	1.95	-0.58	1.02	-0.62	0.76
H2AX_4_11 K5acK9ac	0.63%	8.80%	0.93%	1.80%	0.39%	10.28%	0.19%	1.59%	3.81	1.21	0.95	0.80	0.56	1.15
H2AX_4_11 K9me1	69.02%	40.24%	70.86%	54.09%	1.23%	26.76%	1.13%	16.06%	-0.78	1.65	-0.39	1.60	0.04	2.01
H2AX_4_11 K5me1	24.06%	47.05%	21.66%	34.80%	1.21%	17.80%	1.51%	15.15%	0.97	1.98	0.68	1.32	-0.15	2.30
H2A1_1_11 unmod	50.90%	40.55%	3.13%	9.26%	9.77%	34.81%	0.39%	3.87%	-0.33	0.43	1.56	2.39	-4.02	5.23
H2A1_1_11 S1ac	1.46%	0.33%	0.00%	0.50%	0.49%	0.40%	0.00%	0.52%	-2.15	3.39	0.00	0.00	0.00	0.00
H2A1_1_11 K5ac	47.64%	59.12%	96.87%	90.24%	9.83%	35.20%	0.39%	3.53%	0.31	0.47	-0.10	2.77	1.02	5.30
H2AZ_1_19 unmod	91.49%	90.96%	91.19%	90.54%	1.57%	10.99%	1.08%	2.29%	-0.01	0.06	-0.01	0.38	0.00	0.22
H2AZ_1_19 K4ac	2.59%	4.58%	3.81%	2.79%	0.59%	5.61%	0.15%	0.89%	0.82	0.50	-0.45	2.08	0.55	3.83
H2AZ_1_19 K7ac	4.95%	0.00%	2.68%	3.95%	0.80%	0.00%	0.44%	0.76%	0.00	0.00	0.56	2.71	-0.88	4.72
H2AZ_1_19 K11ac	0.31%	0.04%	1.08%	1.43%	0.37%	0.05%	0.19%	0.36%	-2.79	1.05	0.41	1.53	1.82	3.51
H2AZ_1_19 K15ac	0.01%	4.37%	0.12%	0.34%	0.01%	5.36%	0.05%	0.29%	8.84	1.24	1.45	1.22	3.70	4.17
H2AZ_1_19 K4acK7ac	0.26%	0.00%	0.58%	0.28%	0.17%	0.00%	0.27%	0.18%	0.00	0.00	-1.04	1.61	1.17	1.81
H2AZ_1_19 K4acK11ac	0.24%	0.00%	0.01%	0.11%	0.19%	0.00%	0.01%	0.07%	0.00	0.00	4.38	2.34	-5.45	1.81
H2AZ_1_19 K4acK15ac	0.02%	0.00%	0.00%	0.00%	0.03%	0.00%	0.00%	0.00%	0.00	0.00	0.00	0.00	-2.85	1.06
H2AZ_1_19 K7acK11ac	0.00%	0.00%	0.39%	0.32%	0.00%	0.00%	0.25%	0.17%	0.00	0.00	-0.30	0.37	0.00	0.00
H2AZ_1_19 K7acK15ac	0.00%	0.00%	0.00%	0.05%	0.00%	0.00%	0.00%	0.07%	0.00	0.00	0.00	0.00	0.00	0.00
H2AZ_1_19 K11acK15ac	0.07%	0.00%	0.08%	0.09%	0.06%	0.00%	0.03%	0.09%	0.00	0.00	0.14	0.12	0.26	0.30
H2AZ_1_19 K4acK7acK15ac	0.02%	0.00%	0.04%	0.05%	0.03%	0.00%	0.03%	0.06%	0.00	0.00	0.14	0.09	1.08	0.88
H2AZ_1_19 K4acK7acK11ac	0.03%	0.00%	0.02%	0.05%	0.03%	0.00%	0.01%	0.06%	0.00	0.00	1.66	0.89	-0.62	0.40
H2AZ_1_19 K4acK7acK11acK15ac	0.03%	0.04%	0.00%	0.00%	0.04%	0.05%	0.00%	0.00%	0.52	0.29	0.00	0.00	0.00	0.00
H2A1_12_17 unmod	56.29%	62.92%	48.09%	45.61%	0.44%	0.95%	2.58%	11.96%	0.16	9.38	-0.08	0.29	-0.23	5.75
H2A1_12_17 K13ac	0.00%	0.00%	0.02%	0.02%	0.00%	0.00%	0.01%	0.01%	0.00	0.00	0.15	0.21	2.62	4.88
H2A1_12_17 K15ac	4.10%	0.87%	18.99%	23.61%	1.29%	0.67%	6.49%	13.29%	-2.24	4.24	0.31	0.47	2.21	4.29
H2A1_12_17 K15me1	38.96%	36.18%	30.02%	26.38%	0.91%	0.31%	4.74%	4.84%	-0.11	5.39	-0.19	0.87	-0.38	3.54
H2A1_12_17 K13me1	0.40%	0.00%	2.50%	3.55%	0.21%	0.00%	0.72%	1.20%	0.00	0.00	0.50	1.28	2.65	5.24
H2A1_12_17 T16ac	0.25%	0.03%	0.37%	0.84%	0.16%	0.04%	0.14%	0.51%	-2.86	2.53	1.16	1.53	0.56	0.94
H2A3_12_17 unmod	5.73%	1.27%	19.49%	23.98%	1.62%	0.94%	4.88%	12.82%	-2.17	4.51	0.30	0.50	1.77	5.03
H2A3_12_17 K13ac	0.00%	0.00%	0.00%	0.00%	0.00%	0.00%	0.00%	0.00%	0.00	0.00	0.00	0.00	0.00	0.00
H2A3_12_17 K15ac	0.00%	0.01%	0.00%	0.00%	0.00%	0.02%	0.00%	0.00%	0.00	0.00	0.00	0.00	0.00	0.00
H2A3_12_17 K15me1	14.10%	67.20%	80.20%	44.43%	2.36%	38.23%	4.96%	24.00%	2.25	2.12	-0.85	2.75	2.51	12.98
H2A3_12_17 K13me1	80.18%	31.51%	0.31%	31.59%	3.97%	37.27%	0.18%	36.82%	-1.35	2.00	6.65	1.30	-7.99	9.27
H2A1_72_77 unmod	100.00%	100.00%	99.86%	99.99%	0.00%	0.00%	0.17%	0.01%	0.00	0.00	0.00	1.16	0.00	0.00
H2A1_72_77 K74ac	0.00%	0.00%	0.14%	0.01%	0.00%	0.00%	0.17%	0.01%	0.00	0.00	-3.98	1.16	0.00	0.00
H2A_1_88 H2A14s.HLQLAIR	44.43%	43.52%	63.99%	57.59%	8.29%	12.91%	2.25%	22.37%	-0.03	0.08	-0.15	0.40	0.53	4.34
H2A_1_88 H2AZ.AGGKAGKDSGKAKTKAVSR	53.41%	42.63%	34.15%	40.40%	7.96%	11.92%	2.02%	21.74%	-0.33	1.29	0.24	0.40	-0.65	4.46
H2A_1_88 H2AY.SAKAGVIFPVGR	0.91%	12.70%	0.80%	1.19%	0.26%	4.79%	0.22%	0.45%	3.80	3.40	0.58	1.35	-0.19	0.51
H2A_1_88 H2AX.GKTGGKAR	1.24%	1.15%	1.05%	0.82%	0.28%	0.35%	0.12%	0.33%	-0.12	0.31	-0.35	1.09	-0.24	1.06

Table S3. Effects of MGL50 on PTMs of H2B histone variant in Hs27 and HT-29 cells. Cells were seeded (2.5x10[5] cells/well) into 12-well plates, were treated with 0.03 mg/mL MGL (MGL50) for 48 h and then were collected. Histones were extracted and peptides were analyzed by mass spectrometry. Data are represented as mean and standard error. NT, non-treated; MGL50, concentration of MGL able to reduce cell viability to 50%. Heteroscedastic two-tails t-test are applied and statistical analysis was carried out comparing Hs27 treated vs not treated, HT-29 treated vs not treated, HT-29 non treated vs Hs27 non treated. p-value are reported as -log2. Statistically significant differences are highlighted in green.

	mean				standard error									
	Hs27 cells		HT-29 cells		Hs27 cells		HT-29 cells							
	NT	MGL50	NT	MGL50	NT	MGL50	NT	MGL50	Hs27 MGL50/NT	p-value	HT-29 MGL50/NT	p-value	HT29/Hs27 NT	p-value
H2B1B_80_86 unmod	99.87%	100.00%	99.33%	98.54%	0.16%	0.00%	0.82%	1.59%	0.00	0.00	-0.01	0.69	-0.01	1.07
H2B1B_80_86 Y83ac	0.13%	0.00%	0.67%	1.46%	0.16%	0.00%	0.82%	1.59%	0.00	0.00	1.12	0.69	2.33	1.07
H2B1A_81_87 unmod	26.09%	8.13%	3.32%	2.42%	16.10%	9.96%	4.06%	2.96%	-1.68	1.69	-0.46	0.26	-2.98	2.57
H2B1A_81_87 K86ac	73.91%	25.20%	96.68%	97.58%	16.10%	30.87%	4.06%	2.96%	-1.55	2.63	0.01	0.26	0.39	2.57
H2B_1_29 1C.PEPAKSAPAPKKGSKKAVTKAQKKDGKKR	62.08%	75.39%	46.65%	42.48%	1.31%	5.89%	5.79%	7.06%	0.28	4.21	-0.14	0.72	-0.41	4.91
H2B_1_29 1H.PDPAKSAPAPKKGSKKAVTKAQKKDGKKR	11.11%	8.18%	5.20%	16.68%	0.84%	2.83%	1.20%	10.04%	-0.44	1.78	1.68	1.76	-1.10	7.01
H2B_1_29 2F.PDPAKSAPAPKKGSKKAVTKVQKKDGKKR	2.05%	0.00%	1.12%	3.62%	2.30%	0.00%	1.08%	3.60%	0.00	0.00	1.70	1.12	-0.88	0.57
H2B_1_29 1B.PEPSKSAPAPKKGSKKAITKAQKKDGKKR	2.25%	0.00%	0.08%	0.00%	2.76%	0.00%	0.09%	0.00%	0.00	0.00	0.00	0.00	-4.86	1.20
H2B_1_29 1N.PEPSKSAPAPKKGSKKAVTKAQKKDGKKR	16.96%	1.68%	40.77%	23.37%	7.80%	1.06%	8.77%	17.52%	-3.34	2.88	-0.80	1.56	1.27	3.88
H2B_1_29 1D.PEPTKSAPAPKKGSKKAVTKAQKKDGKKR	5.54%	3.95%	4.04%	6.89%	0.88%	2.62%	4.95%	4.79%	-0.49	0.95	0.77	0.65	-0.46	0.45
H2B_1_29 1M.PEPVKSAPVPKKGSKKAINKAQKKDGKKR	0.00%	0.00%	1.46%	0.68%	0.00%	0.00%	1.79%	0.52%	0.00	0.00	-1.11	0.66	0.00	0.00
H2B_1_29 1L.PELAKSAPAPKKGSKKAVTKAQKKDGKKR	0.00%	10.81%	0.68%	6.27%	0.00%	4.12%	0.84%	7.69%	0.00	0.00	3.20	1.10	0.00	0.00

Table S4. Effects of MGL50 on PTMs of H3.3 histone variant in Hs27 and HT-29 cells. Cells were seeded (2.5x10^5 cells/well) into 12-well plates, were treated with 0.03 mg/mL MGL (MGL50) for 48 h and then were collected. Histones were extracted and peptides were analyzed by mass spectrometry. Data are represented as mean and standard error. NT, non-treated; MGL50, concentration of MGL able to reduce cell viability to 50%. Heteroscedastic two-tails t-test are applied and statistical analysis was carried out comparing Hs27 treated vs not treated, HT-29 treated vs not treate,; HT-29 non treated vs Hs27 non treated. p-value are reported as -log2. Statistically significant differences are highlighted in green.

	mean				standard error									
	Hs27 cells		HT-29 cells		Hs27 cells		HT-29 cells							
	NT	MGL50	NT	MGL50	NT	MGL50	NT	MGL50	Hs27 MGL50/NT	p-value	HT-29 MGL50/NT	p-value	HT29/Hs27 NT	p-value
H33_27_40 unmod	0.55%	4.12%	0.22%	6.04%	0.67%	5.05%	0.23%	3.96%	2.91	1.06	4.80	2.23	-1.34	0.75
H33_27_40 K36me1	0.20%	0.00%	0.00%	0.36%	0.25%	0.00%	0.00%	0.44%	0.00	0.00	0.00	0.00	0.00	0.00
H33_27_40 K27me1	0.18%	0.00%	0.00%	1.08%	0.22%	0.00%	0.00%	1.17%	0.00	0.00	0.00	0.00	0.00	0.00
H33_27_40 K27me2	4.87%	1.19%	13.33%	13.36%	3.20%	1.46%	2.12%	5.22%	-2.03	1.89	0.00	0.01	1.45	4.21
H33_27_40 K36me2	7.76%	4.22%	3.98%	3.63%	3.05%	5.17%	1.22%	2.23%	-0.88	0.97	-0.13	0.19	-0.96	2.12
H33_27_40 K27me3	0.74%	0.00%	5.17%	5.67%	0.91%	0.00%	0.88%	2.47%	0.00	0.00	0.13	0.27	2.80	6.28
H33_27_40 K36me3	4.09%	2.15%	1.08%	0.80%	0.92%	2.63%	0.23%	0.17%	-0.93	1.18	-0.44	1.74	-1.92	5.83
H33_27_40 K27me2K36me1	9.19%	4.03%	9.55%	8.98%	3.12%	4.93%	1.62%	4.05%	-1.19	1.56	-0.09	0.18	0.06	0.14
H33_27_40 K27me1K36me2	13.23%	10.60%	5.41%	5.85%	2.30%	9.26%	0.92%	1.69%	-0.32	0.41	0.11	0.33	-1.29	5.79
H33_27_40 K27me1K36me1	2.16%	0.33%	0.02%	0.44%	1.98%	0.40%	0.02%	0.23%	-2.74	1.61	4.56	2.77	-6.85	1.66
H33_27_40 K27me3K36me1	0.00%	0.00%	1.10%	1.67%	0.00%	0.00%	0.68%	1.03%	0.00	0.00	0.60	0.73	0.00	0.00
H33_27_40 K27me1K36me3	4.62%	1.36%	1.57%	2.93%	0.90%	1.66%	1.07%	2.37%	-1.77	3.29	0.90	0.84	-1.56	4.17
H33_27_40 K27me2K36me2	11.72%	7.53%	12.45%	14.86%	2.24%	9.22%	1.47%	6.88%	-0.64	0.70	0.26	0.52	0.09	0.41
H33_27_40 K27me3K36me2	2.19%	0.00%	2.80%	2.85%	0.98%	0.00%	0.65%	1.77%	0.00	0.00	0.02	0.03	0.35	0.84

Table S5. Effects of MGL50 on PTMs of H1 histone variant in HT-29 cells. Cells were seeded ($2.5x10^5$ cells/well) into 12-well plates, were treated with 0.03 mg/mL MGL (MGL50) for 24, 48 and 72 h and then were collected. Histones were extracted and peptides were analyzed by mass spectrometry. Data are represented as mean and standard deviation. NT, non-treated; PLP, control vehicle, cells treated with 0.5 mM PLP in PBS; MGL50, concentration of MGL able to reduce cell viability to 50%. Heteroscedastic two-tails t-test are applied and statistical analysis was carried out comparing, at each incubation time, NT vs PLP and PLP vs MGL. *= $p \leq 0.05$; **= $p \leq 0.01$ and ***= $p \leq 0.001$. Statistically significant differences are highlighted in green.

H1 variant	mean								
	NT 24h	NT 48h	NT 72h	PLP 24h	PLP 48h	PLP 72h	MGL50 24h	MGL50 48h	MGL50 72h
H14_25_32 unmod	82.64%	77.83%	83.52%	77.67%	76.57%	84.73%	71.90%	84.77%	83.23%
H14_25_32 K25me1	4.83%	9.69%	1.75%	8.78%	12.17%	1.70%	14.75%	2.31%	5.31%
H14_25_32 K25me2	0.60%	0.80%	0.15%	0.65%	0.63%	0.31%	0.43%	0.20%	0.05%
H14_25_32 K25me3	0.00%	0.00%	0.00%	0.00%	0.00%	0.00%	0.00%	0.00%	0.00%
H14_25_32 K25ac	0.75%	0.92%	1.05%	0.45%	0.84%	0.83%	0.89%	0.02%	0.66%
H14_25_32 K31ac	8.46%	7.58%	7.70%	6.21%	6.34%	10.49%	7.91%	10.52%	8.74%
H14_25_32 S26ac	1.83%	1.07%	2.26%	3.55%	1.87%	0.82%	1.46%	0.78%	0.53%
H14_25_32 S26ph	0.90%	2.12%	3.57%	2.69%	1.59%	1.12%	2.66%	1.41%	1.48%
H12_33_53 unmod	68.60%	72.95%	70.05%	65.05%	62.57%	67.78%	71.37%	56.49%	58.42%
H12_33_53 K33me1	29.49%	25.66%	29.32%	30.20%	34.43%	31.56%	25.66%	8.90%	19.49%
H12_33_53 K33me2	0.03%	0.28%	0.35%	0.48%	1.19%	0.40%	2.21%	0.52%	0.66%
H12_33_53 K33me3	1.06%	0.22%	0.15%	2.59%	0.13%	0.01%	0.05%	15.87%	16.20%
H12_33_53 K33ac	0.45%	0.87%	0.12%	0.00%	0.84%	0.25%	0.36%	18.22%	2.72%
H12_33_53 S40ac	0.38%	0.02%	0.01%	1.69%	0.83%	0.00%	0.36%	0.00%	2.52%
H15_36_56 unmod	27.07%	25.49%	28.98%	27.76%	26.49%	31.59%	23.26%	7.67%	19.29%
H15_36_56 K36me1	0.02%	0.00%	0.14%	0.00%	1.46%	0.03%	0.00%	1.21%	0.00%
H15_36_56 K36me2	0.21%	0.22%	0.15%	1.97%	0.18%	0.01%	0.42%	33.33%	17.39%
H15_36_56 K36me3	0.92%	1.52%	0.70%	1.92%	0.89%	0.06%	6.25%	0.96%	0.00%
H15_36_56 K36ac	67.16%	72.33%	69.26%	67.94%	69.47%	67.82%	70.07%	56.61%	59.02%
H15_36_56 S43ac	4.61%	0.44%	0.78%	0.41%	1.51%	0.48%	0.00%	0.20%	4.30%
H1_1_35 H12.SETAPAAPAAAPPAEKAPVKKKAAKKA	0.72%	10.96%	0.17%	1.10%	1.39%	0.00%	11.98%	0.00%	1.81%
H1_1_35 H13.SETAPLAPTIPAPAEKTPVKKKAKKAGAT	29.11%	29.08%	5.37%	20.55%	1.42%	1.75%	11.93%	30.57%	34.79%
H1_1_35 H14.SETAPAAPAAPAPAEKTPVKKKAR	55.20%	19.08%	21.21%	58.22%	66.80%	14.77%	22.79%	54.93%	29.11%
H1_1_35 H15.SETAPAETATPAPVEKSPAKKKATKKAA	14.98%	40.88%	73.25%	20.12%	30.40%	83.48%	53.30%	14.50%	34.30%
H1_54_81 H11.GGVSLAALKKALAAAGYDVEKNNSR	3.64%	25.27%	10.36%	0.00%	4.46%	0.22%	17.61%	53.82%	60.96%
H1_54_81 H1v234.SGVSLAALKKALAAAGYDVEKNNS	58.46%	44.85%	60.62%	61.80%	69.93%	60.83%	59.05%	38.49%	29.02%
H1_54_81 H15.NGLSLAALKKALAAGGYDVEKNNSR	37.83%	29.64%	28.97%	37.99%	24.56%	38.54%	22.80%	7.69%	10.02%
H1_54_81 H1T.VGMSLVALKKALAAAGYDVEKNNSR	0.07%	0.23%	0.04%	0.22%	1.05%	0.41%	0.54%	0.00%	0.00%

H1 variant	standard deviation									NT vs PLP			PLP vs MGL		
	NT 24h	NT 48h	NT 72h	PLP 24h	PLP 48h	PLP 72h	MGL50 24h	MGL50 48h	MGL50 72h	24h	48h	72h	24h	48h	72h
H14_25_32 unmod	0.05	0.06	0.02	0.14	0.19	0.02	0.11	0.01	0.02	0.60	0.92	0.42	0.613	0.525	0.440
H14_25_32 K25me1	0.06	0.08	0.01	0.13	0.18	0.00	0.12	0.00	0.03	0.65	0.84	0.87	0.579	0.444	0.143
H14_25_32 K25me2	0.00	0.00	0.00	0.01	0.00	0.00	0.00	0.00	0.00	0.88	0.54	0.23	0.521	0.015	0.069
H14_25_32 K25me3	0.00	0.00	0.00	0.00	0.00	0.00	0.00	0.00	0.00	1.00	1.00	1.00	1.000	1.000	1.000
H14_25_32 K25ac	0.00	0.01	0.01	0.01	0.01	0.01	0.01	0.00	0.01	0.52	0.91	0.77	0.458	0.203	0.787
H14_25_32 K31ac	0.01	0.03	0.02	0.02	0.02	0.00	0.01	0.01	0.01	0.16	0.59	0.04	0.305	0.030	0.030
H14_25_32 S26ac	0.01	0.00	0.01	0.01	0.00	0.00	0.00	0.00	0.00	0.20	0.05	0.10	0.127	0.024	0.369
H14_25_32 S26ph	0.00	0.00	0.01	0.01	0.00	0.01	0.01	0.00	0.01	0.06	0.23	0.05	0.965	0.591	0.576
H12_33_53 unmod	0.04	0.06	0.03	0.03	0.11	0.02	0.03	0.50	0.10	0.31	0.21	0.31	0.050	0.847	0.170
H12_33_53 K33me1	0.02	0.04	0.02	0.04	0.08	0.01	0.05	0.13	0.14	0.78	0.16	0.24	0.286	0.040	0.282
H12_33_53 K33me2	0.00	0.00	0.01	0.00	0.01	0.01	0.04	0.00	0.01	0.25	0.21	0.92	0.494	0.330	0.744
H12_33_53 K33me3	0.01	0.00	0.00	0.03	0.00	0.00	0.00	0.27	0.14	0.50	0.65	0.19	0.314	0.426	0.183
H12_33_53 K33ac	0.01	0.01	0.00	0.00	0.01	0.00	0.00	0.30	0.05	1.00	0.98	0.41	1.000	0.416	0.459
H12_33_53 S40ac	0.01	0.00	0.00	0.02	0.01	0.00	0.00	0.00	0.04	0.24	0.43	0.68	0.218	1.000	0.423
H15_36_56 unmod	0.02	0.05	0.02	0.01	0.03	0.02	0.04	0.11	0.13	0.66	0.78	0.19	0.141	0.044	0.232
H15_36_56 K36me1	0.00	0.00	0.00	0.00	0.02	0.00	0.00	0.02	0.00	1.00	1.00	0.45	1.000	0.888	1.000
H15_36_56 K36me2	0.00	0.00	0.00	0.03	0.00	0.00	0.01	0.58	0.15	0.47	0.86	0.24	0.485	0.425	0.175
H15_36_56 K36me3	0.01	0.02	0.01	0.02	0.02	0.00	0.05	0.02	0.00	0.42	0.71	0.19	0.222	0.960	1.000
H15_36_56 K36ac	0.03	0.03	0.03	0.02	0.05	0.02	0.03	0.49	0.05	0.72	0.47	0.48	0.366	0.697	0.040
H15_36_56 S43ac	0.04	0.01	0.01	0.00	0.02	0.00	0.00	0.00	0.07	0.20	0.43	0.65	1.000	0.326	0.468
H1_1_35 H12.SETAPAAPAAAPPAEKAPVKKKAAKKA	0.00	0.14	0.00	0.02	0.02	0.00	0.11	0.00	0.03	0.76	0.31	1.00	0.163	1.000	1.000
H1_1_35 H13.SETAPLAPTIPAPAEKTPVKKKAKKAGAT	0.39	0.48	0.07	0.28	0.01	0.03	0.21	0.34	0.22	0.77	0.43	0.46	0.691	0.276	0.115
H1_1_35 H14.SETAPAAPAAPAPAEKTPVKKKAR	0.48	0.33	0.22	0.50	0.48	0.26	0.39	0.48	0.50	0.94	0.23	0.76	0.392	0.777	0.683
H1_1_35 H15.SETAPAETATPAPVEKSPAKKKATKKAA	0.12	0.44	0.24	0.24	0.51	0.24	0.45	0.16	0.31	0.75	0.80	0.63	0.320	0.633	0.094
H1_54_81 H11.GGVSLAALKKALAAAGYDVEKNNSR	0.06	0.43	0.18	0.00	0.07	0.00	0.31	0.48	0.33	1.00	0.49	0.43	1.000	0.215	0.084
H1_54_81 H1v234.SGVSLAALKKALAAAGYDVEKNNS	0.04	0.28	0.20	0.18	0.16	0.10	0.13	0.53	0.17	0.77	0.25	0.99	0.842	0.382	0.049
H1_54_81 H15.NGLSLAALKKALAAGGYDVEKNNSR	0.04	0.16	0.02	0.18	0.13	0.11	0.17	0.13	0.17	0.99	0.69	0.20	0.343	0.196	0.072
H1_54_81 H1T.VGMSLVALKKALAAAGYDVEKNNSR	0.00	0.00	0.00	0.00	0.02	0.01	0.01	0.00	0.00	0.35	0.44	0.34	0.592	1.000	1.000

Table S6. Effects of MGL50 on PTMs of H2A histone variant in HT-29 cells. Cells were seeded ($2.5x10^5$ cells/well) into 12-well plates, were treated with 0.03 mg/mL MGL (MGL50) for 24, 48 and 72 h and then were collected. Histones were extracted and peptides were analyzed by mass spectrometry. Data are represented as mean and standard deviation. NT, non-treated; PLP, control vehicle, cells treated with 0.5 mM PLP in PBS; MGL50, concentration of MGL able to reduce cell viability to 50%. Heteroscedastic two-tails t-test are applied and statistical analysis was carried out comparing, at each incubation time, NT vs PLP and PLP vs MGL. *= p ≤ 0.05; **= p ≤ 0.01 and ***= p ≤ 0.001. Statistically significant differences are highlighted in green.

H2A variant	mean									standard deviation									NT vs PLP			PLP vs MGL		
	NT 24h	NT 48h	NT 72h	PLP 24h	PLP 48h	PLP 72h	MGL50 24	MGL50 48	MGL50 72	NT 24h	NT 48h	NT 72h	PLP 24h	PLP 48h	PLP 72h	MGL50 24h	MGL50 48h	MGL50 72	24h	48h	72h	24h	48h	72h
H2A1_36_42 unmod	99.87%	99.81%	99.91%	99.91%	99.84%	99.92%	99.88%	99.83%	99.84%	0.00	0.00	0.00	0.00	0.00	0.00	0.00	0.00	0.00	0.15	0.65	0.76	0.246	0.880	0.053
H2A1_36_42 K36ac	0.12%	0.16%	0.05%	0.09%	0.14%	0.07%	0.10%	0.13%	0.16%	0.00	0.00	0.00	0.00	0.00	0.00	0.00	0.00	0.00	0.16	0.76	0.21	0.579	0.853	0.038
H2A1_36_42 Y39ac	0.00%	0.03%	0.05%	0.00%	0.02%	0.01%	0.02%	0.03%	0.00%	0.00	0.00	0.00	0.00	0.00	0.00	0.00	0.00	0.00	1.00	0.71	1.00	1.000	0.251	1.000
H2A3_36_42 unmod	99.97%	99.28%	99.69%	99.58%	99.06%	99.86%	99.50%	99.20%	99.09%	0.00	0.00	0.00	0.00	0.01	0.00	0.00	0.00	0.01	0.08	0.71	0.34	0.788	0.820	0.222
H2A3_36_42 K36ac	0.00%	0.06%	0.00%	0.01%	0.05%	0.00%	0.00%	0.01%	0.25%	0.00	0.00	0.00	0.00	0.00	0.00	0.00	0.00	0.00	0.52	0.83	1.00	1.000	0.482	0.428
H2A3_36_42 Y39ac	0.02%	0.66%	0.31%	0.41%	0.89%	0.14%	0.50%	0.80%	0.67%	0.00	0.00	0.00	0.00	0.01	0.00	0.00	0.00	0.01	0.14	0.68	0.34	0.779	0.868	0.434
H2AX_36_42 unmod	99.92%	99.26%	99.97%	98.81%	99.63%	99.91%	98.61%	99.72%	99.52%	0.00	0.00	0.00	0.02	0.00	0.00	0.01	0.00	0.01	0.37	0.15	0.50	0.872	0.684	0.505
H2AX_36_42 K36ac	0.02%	0.11%	0.00%	0.02%	0.11%	0.00%	0.00%	0.13%	0.48%	0.00	0.00	0.00	0.00	0.00	0.00	0.00	0.00	0.01	0.80	0.98	1.00	0.477	0.885	1.000
H2AX_36_42 Y39ac	0.06%	0.63%	0.03%	1.17%	0.26%	0.09%	1.39%	0.15%	0.00%	0.00	0.00	0.00	0.02	0.00	0.00	0.01	0.00	0.00	0.37	0.07	0.47	0.864	0.588	1.000
H2A1_4_11 unmod	93.04%	89.05%	87.24%	89.44%	89.88%	92.95%	92.13%	95.43%	95.84%	0.00	0.03	0.06	0.02	0.03	0.02	0.02	0.00	0.01	0.10	0.73	0.19	0.184	0.061	0.092
H2A1_4_11 K5ac	2.41%	1.00%	1.99%	0.98%	2.00%	3.29%	2.47%	1.74%	2.06%	0.02	0.02	0.02	0.02	0.02	0.00	0.00	0.02	0.02	0.41	0.52	0.34	0.210	0.853	0.360
H2A1_4_11 K9ac	1.60%	2.61%	1.34%	2.39%	1.56%	0.48%	0.58%	1.23%	1.20%	0.02	0.02	0.02	0.02	0.02	0.00	0.00	0.02	0.01	0.63	0.51	0.43	0.223	0.813	0.491
H2A1_4_11 K5acK9ac	0.09%	0.17%	0.06%	0.10%	0.14%	0.13%	0.15%	0.26%	0.05%	0.00	0.00	0.00	0.00	0.00	0.00	0.00	0.00	0.00	0.89	0.68	0.39	0.453	0.154	0.343
H2A1_4_11 K9me1	0.41%	2.71%	3.73%	2.08%	2.25%	1.17%	1.80%	0.44%	0.49%	0.00	0.01	0.04	0.01	0.02	0.01	0.02	0.00	0.00	0.05	0.76	0.33	0.842	0.195	0.252
H2A1_4_11 K5me1	2.44%	4.47%	5.63%	5.02%	4.17%	1.97%	2.87%	0.91%	0.36%	0.00	0.02	0.02	0.02	0.01	0.01	0.02	0.01	0.00	0.15	0.84	0.08	0.287	0.023	0.157
H2AJ_4_11 unmod	11.82%	16.07%	22.70%	14.65%	16.58%	13.98%	14.61%	6.17%	5.31%	0.00	0.03	0.06	0.04	0.04	0.05	0.02	0.01	0.04	0.30	0.86	0.11	0.986	0.010	0.066
H2AJ_4_11 K5ac	0.00%	0.01%	0.00%	0.00%	0.64%	0.01%	0.01%	0.00%	0.00%	0.00	0.00	0.00	0.00	0.01	0.00	0.00	0.00	0.00	1.00	0.42	1.00	1.000	1.000	1.000
H2AJ_4_11 K9ac	1.81%	4.16%	3.30%	3.71%	2.54%	1.83%	2.32%	1.02%	0.21%	0.00	0.01	0.01	0.01	0.02	0.01	0.01	0.00	0.00	0.03	0.27	0.17	0.090	0.294	0.068
H2AJ_4_11 K5acK9ac	59.14%	53.72%	37.13%	50.24%	47.93%	57.24%	47.57%	74.63%	57.81%	0.03	0.09	0.16	0.13	0.13	0.11	0.06	0.07	0.50	0.32	0.56	0.14	0.772	0.036	0.986
H2AJ_4_11 K9me1	4.74%	9.72%	5.44%	6.47%	1.63%	5.89%	1.42%	0.03%	0.04%	0.08	0.08	0.07	0.03	0.03	0.02	0.02	0.00	0.00	0.74	0.16	0.92	0.070	0.423	0.031
H2AJ_4_11 K5me1	22.47%	16.32%	31.43%	24.93%	30.67%	21.05%	34.08%	18.15%	36.64%	0.06	0.04	0.04	0.11	0.08	0.05	0.06	0.05	0.46	0.75	0.05	0.05	0.275	0.086	0.617
H2AX_4_11 unmod	9.97%	9.60%	40.93%	8.49%	8.94%	11.32%	10.79%	10.93%	10.11%	0.01	0.02	0.17	0.01	0.04	0.01	0.00	0.01	0.01	0.20	0.79	0.10	0.051	0.402	0.179
H2AX_4_11 K5ac	0.16%	0.41%	1.82%	0.34%	0.24%	0.43%	0.24%	0.32%	0.17%	0.00	0.00	0.01	0.00	0.00	0.00	0.00	0.00	0.00	0.02	0.05	0.22	0.193	0.271	0.169
H2AX_4_11 K9ac	0.47%	0.75%	2.35%	0.53%	0.50%	0.56%	0.75%	0.75%	0.29%	0.00	0.00	0.00	0.00	0.01	0.00	0.00	0.00	0.00	0.84	0.52	0.003978	0.412	0.542	0.247
H2AX_4_11 K5acK9ac	0.06%	0.25%	0.58%	0.07%	0.09%	0.05%	0.08%	0.05%	0.01%	0.00	0.00	0.00	0.00	0.00	0.00	0.00	0.00	0.00	0.63	0.21	0.003809	0.868	0.206	0.070
H2AX_4_11 K9me1	42.37%	41.93%	37.99%	63.37%	63.62%	68.41%	0.09%	42.39%	46.00%	0.37	0.36	0.14	0.02	0.02	0.10	0.00	0.37	0.40	0.43	0.41	0.037896	0.000	0.421	0.396
H2AX_4_11 K5me1	46.97%	47.05%	16.33%	27.19%	26.60%	19.24%	88.05%	45.55%	43.42%	0.36	0.37	0.05	0.00	0.01	0.09	0.00	0.37	0.39	0.44	0.44	0.65	0.000	0.464	0.354
H2A1_1_11 unmod	20.16%	35.49%	30.86%	30.60%	39.57%	40.87%	17.92%	31.39%	18.99%	0.03	0.02	0.05	0.09	0.03	0.06	0.04	0.09	0.06	0.13	0.11	0.09	0.086	0.214	0.011
H2A1_1_11 S1ac	6.91%	19.53%	25.08%	25.33%	28.87%	10.64%	12.81%	14.80%	13.43%	0.05	0.07	0.09	0.15	0.10	0.02	0.04	0.06	0.02	0.11	0.26	0.06	0.234	0.104	0.162
H2A1_1_11 K5ac	72.93%	44.98%	44.06%	44.07%	31.57%	48.49%	69.27%	53.81%	67.58%	0.07	0.08	0.09	0.23	0.13	0.08	0.07	0.15	0.05	0.11	0.20	0.55	0.144	0.121	0.024
H2AV_1_19 unmod	85.85%	86.79%	89.48%	83.56%	80.90%	87.46%	82.35%	79.55%	85.34%	0.02	0.06	0.01	0.08	0.07	0.01	0.09	0.06	0.13	0.64	0.33	0.03	0.868	0.816	0.808
H2AV_1_19 K4ac	0.08%	1.51%	1.79%	1.08%	0.56%	1.05%	1.21%	0.00%	0.00%	0.00	0.02	0.01	0.00	0.00	0.01	0.01	0.00	0.00	0.01	0.38	0.42	0.875	1.000	1.000
H2AV_1_19 K7ac	0.09%	0.17%	1.98%	0.12%	1.08%	4.28%	0.32%	0.00%	0.00%	0.00	0.00	0.01	0.00	0.02	0.02	0.01	0.00	0.00	0.81	0.49	0.13	0.597	1.000	1.000
H2AV_1_19 K11ac	1.77%	0.04%	2.86%	1.87%	0.54%	1.22%	2.21%	0.05%	0.01%	0.02	0.00	0.01	0.02	0.01	0.01	0.02	0.00	0.00	0.96	0.26	0.10	0.855	0.266	0.076
H2AV_1_19 K15ac	2.46%	4.29%	1.58%	3.17%	6.93%	2.80%	5.17%	4.52%	0.87%	0.01	0.02	0.01	0.01	0.05	0.01	0.05	0.07	0.02	0.38	0.45	0.10	0.557	0.644	0.119
H2AV_1_19 K4acK7ac	0.12%	0.00%	0.04%	0.00%	0.00%	0.00%	0.00%	0.00%	0.00%	0.00	0.00	0.00	0.00	0.00	0.00	0.00	0.00	0.00	1.00	1.00	1.00	1.000	1.000	1.000
H2AV_1_19 K4acK11ac	0.00%	0.00%	0.00%	0.00%	0.00%	0.00%	0.00%	0.00%	0.00%	0.00	0.00	0.00	0.00	0.00	0.00	0.00	0.00	0.00	1.00	1.00	1.00	1.000	1.000	1.000
H2AV_1_19 K4acK15ac	0.19%	0.00%	0.08%	0.02%	0.00%	0.00%	0.03%	0.00%	0.00%	0.00	0.00	0.00	0.00	0.00	0.00	0.00	0.00	0.00	0.16	1.00	1.00	0.678	1.000	1.000
H2AV_1_19 K7acK11ac	1.13%	0.18%	0.30%	0.95%	0.26%	0.68%	0.69%	0.02%	0.00%	0.01	0.00	0.00	0.00	0.00	0.00	0.00	0.00	0.00	0.79	0.60	0.19	0.176	0.247	1.000
H2AV_1_19 K7acK15ac	0.01%	0.00%	0.05%	0.01%	0.01%	0.01%	0.01%	0.00%	0.00%	0.00	0.00	0.00	0.00	0.00	0.00	0.00	0.00	0.00	1.00	0.10	0.41	1.000	0.101	1.000
H2AV_1_19 K11acK15ac	1.60%	0.40%	0.98%	1.23%	1.34%	0.76%	0.77%	0.45%	0.00%	0.02	0.00	0.01	0.01	0.00	0.00	0.00	0.01	0.00	0.73	0.04	0.61	0.358	0.095	1.000
H2AV_1_19 K7acK11acK15ac	0.02%	0.66%	0.29%	0.14%	0.01%	0.04%	0.25%	0.00%	0.00%	0.00	0.00	0.00	0.00	0.00	0.00	0.00	0.00	0.00	0.35	0.14	0.32	0.583	1.000	1.000
H2AV_1_19 K4acK11acK15ac	0.00%	0.02%	0.00%	0.00%	0.00%	0.00%	0.02%	0.00%	0.00%	0.00	0.00	0.00	0.00	0.00	0.00	0.00	0.00	0.00	1.00	1.00	1.00	1.000	1.000	1.000
H2AV_1_19 K4acK7acK15ac	0.23%	0.02%	0.00%	0.75%	0.02%	0.04%	0.02%	0.00%	0.00%	0.00	0.00	0.00	0.01	0.00	0.00	0.00	0.00	0.00	0.53	0.88	1.00	0.413	1.000	1.000
H2AV_1_19 K4acK7acK11ac	0.09%	0.08%	0.05%	0.53%	0.63%	0.08%	0.04%	0.00%	0.00%	0.00	0.00	0.00	0.01	0.00	0.00	0.00	0.00	0.00	0.20	0.43	0.35	0.212	1.000	1.000
H2AV_1_19 K4acK7acK11acK15ac	6.36%	5.84%	0.52%	6.58%	7.71%	1.59%	6.91%	15.42%	13.78%	0.05	0.02	0.00	0.06	0.04	0.01	0.06	0.11	0.12	0.96	0.54	0.14	0.951	0.317	0.225
H2AZ_1_19 unmod	84.83%	87.15%	88.99%	85.42%	86.45%	87.77%	85.14%	88.33%	88.11%	0.03	0.02	0.00	0.00	0.02	0.01	0.02	0.06	0.10	0.77	0.68	0.15	0.807	0.639	0.960
H2AZ_1_19 K4ac	0.62%	1.50%	2.53%	1.13%	3.04%	1.94%	2.82%	2.14%	4.83%	0.00	0.02	0.02	0.00	0.01	0.01	0.01	0.03	0.05	0.16	0.27	0.69	0.072	0.611	0.367
H2AZ_1_19 K7ac	1.23%	0.38%	1.76%	2.46%	1.91%	1.44%	0.83%	1.05%	0.79%	0.01	0.01	0.02	0.01	0.01	0.02	0.00	0.01	0.01	0.10	0.03	0.84	0.038	0.387	0.648
H2AZ_1_19 K11ac	5.84%	4.90%	2.79%	4.48%	2.93%	5.05%	5.69%	2.06%	1.46%	0.00	0.01	0.02	0.00	0.01	0.03	0.01	0.01	0.02	0.00	0.04	0.28	0.187	0.208	0.133
H2AZ_1_19 K15ac	1.67%	2.15%	1.53%	1.73%	1.44%	1.07%	0.85%	3.78%	1.76%	0.02	0.01	0.02	0.01	0.01	0.01	0.01	0.03	0.02	0.95	0.37	0.73	0.234	0.243	0.561
H2AZ_1_19 K4acK7ac	1.49%	0.62%	0.80%	1.75%	1.15%	0.80%	1.02%	0.09%	0.00%	0.00	0.00	0.00	0.00	0.00	0.00	0.00	0.00	0.00	0.03	0.17	0.98	0.108	0.023	1.000
H2AZ_1_19 K4acK11ac	0.35%	0.29%	0.05%	0.10%	0.17%	0.15%	0.22%	0.00%	0.16%	0.00	0.00	0.00	0.00	0.00	0.00	0.00	0.00	0.00	0.30	0.64	0.29	0.446	1.000	0.930
H2AZ_1_19 K4acK15ac	0.08%	0.08%	0.09%	0.13%	0.16%	0.01%	0.09%	0.00%	0.00%	0.00	0.00	0.00	0.00	0.00	0.00	0.00	0.00	0.00	0.44	0.05	1.00	0.582	1.000	1.000
H2AZ_1_19 K7acK11ac	0.82%	1.02%	0.52%	0.89%	1.10%	1.13%	1.50%	1.58%	1.60%	0.00	0.00	0.00	0.00	0.00	0.00	0.01	0.01	0.02	0.76	0.83	0.02	0.177	0.509	0.631
H2AZ_1_19 K7acK15ac	0.17%	0.01%	0.16%	0.10%	0.02%	0.04%	0.03%	0.01%	0.02%	0.00	0.00	0.00	0.00	0.00	0.00	0.00	0.00	0.00	0.38	0.10	0.30	0.034	0.152	0.666
H2AZ_1_19 K11acK15ac	0.10%	0.49%	0.38%	0.19%	0.21%	0.07%	0.24%	0.69%	0.86%	0.00	0.00	0.00	0.00	0.00	0.00	0.00	0.00	0.01	0.45	0.04	0.00	0.726	0.075	0.253
H2AZ_1_19 K7acK11acK15ac	0.00%	0.01%	0.00%	0.00%	0.00%	0.00%	0.00%	0.00%	0.00%	0.00	0.00	0.00	0.00	0.00	0.00	0.00	0.00	0.00	1.00	1.00	1.00	1.000	1.000	1.000
H2AZ_1_19 K4acK11acK15ac	0.00%	0.10%	0.02%	0.03%	0.07%	0.00%	0.02%	0.00%	0.00%	0.00	0.00	0.00	0.00	0.00	0.00	0.00	0.00	0.00	1.00	0.58	0.46	0.738	1.000	1.000
H2AZ_1_19 K4acK7acK15ac	0.08%	0.04%	0.02%	0.09%	0.06%	0.01%	0.09%	0.01%	0.00%	0.00	0.00	0.00	0.00	0.00	0.00	0.00	0.00	0.00	0.92	0.47	1.00	0.956	0.168	1.000
H2AZ_1_19 K4acK7acK11ac	2.46%	0.86%	0.34%	1.13%	0.97%	0.51%	1.12%	0.27%	0.39%	0.02	0.00	0.00	0.00	0.00	0.00	0.00	0.00	0.00	0.23	0.48	0.37	0.983	0.016	0.591
H2AZ_1_19 K4acK7acK11acK15ac	0.26%	0.38%	0.03%	0.36%	0.31%	0.01%	0.34%	0.00%	0.00%	0.00	0.00	0.00	0.00	0.00	0.00	0.00	0.00	0.00	0.58	0.65	0.25	0.941	1.000	1.000
H2A1_12_17 unmod	59.33%	63.94%	57.48%	58.49%	59.66%	55.12%	59.46%	44.12%	38.42%	0.00	0.04	0.02	0.04	0.00	0.00	0.03	0.07	0.06	0.78	0.21	0.08	0.778	0.067	0.035
H2A1_12_17 K13ac	0.03%	0.03%	0.04%	0.05%	0.03%	0.04%	0.03%	0.03%	0.02%	0.00	0.00	0.00	0.00	0.00	0.00	0.00	0.00	0.00	0.12	0.64	0.77	0.025	0.643	0.013
H2A1_12_17 K15ac	26.31%	23.13%	32.11%	25.91%	22.34%	35.36%	24.37%	27.04%	17.41%	0.04	0.02	0.02	0.04	0.03	0.01	0.04	0.04	0.05	0.91	0.75	0.05	0.679	0.207	0.005
H2A1_12_17 K15me1	12.16%	7.49%	6.81%	13.49%	16.18%	5.43%	13.50%	27.57%	41.00%	0.05	0.05	0.01	0.02	0.04	0.01	0.02	0.04	0.09	0.68	0.08	0.16	0.992	0.023	0.021
H2A1_12_17 K13me1	1.41%	4.42%	1.91%	1.47%	0.99%	2.22%	1.35%	0.55%	1.43%	0.01	0.04	0.01	0.01	0.01	0.00	0.00	0.00	0.01	0.91	0.21	0.46	0.774	0.364	0.158
H2A1_12_17 T16ac	0.75%	0.99%	1.64%	0.59%	0.81%	1.83%	1.29%	0.69%	1.72%	0.00	0.00	0.00	0.00	0.00	0.01	0.00	0.01	0.01	0.64	0.49	0.67	0.104	0.756	0.827
H2A3_12_17 unmod	29.94%	26.07%	34.28%	28.39%	26.44%	35.90%	28.46%	32.83%	29.70%	0.04	0.03	0.02	0.02	0.03	0.02	0.05	0.04	0.04	0.56	0.89	0.42	0.981	0.078	0.088
H2A3_12_17 K13ac	0.00%	0.02%	0.01%	0.03%	0.00%	0.01%	0.00%	0.02%	0.00%	0.00	0.00	0.00	0.00	0.00	0.00	0.00	0.00	0.00	0.47	0.05	0.72	0.474	0.067	1.000
H2A3_12_17 K15ac	0.02%	0.01%	0.03%	0.02%	0.03%	0.07%	0.02%	0.09%	0.01%	0.00	0.00	0.00	0.00	0.00	0.00	0.00	0.00	0.00	1.00	0.07	0.47	0.815	0.013	0.374
H2A3_12_17 K15me1	44.14%	70.28%	21.00%	23.27%	22.54%	41.85%	45.71%	0.07%	67.64%	0.38	0.02	0.36	0.40	0.39	0.36	0.40	0.00	0.03	0.55	0.17	0.52	0.529	0.422	0.343
H2A3_12_17 K13me1	25.72%	3.60%	44.64%	48.26%	50.95%	22.16%	25.79%	66.97%	2.60%	0.41	0.01	0.39	0.39	0.41	0.37	0.39	0.04	0.02	0.53	0.19	0.51	0.524	0.572	0.454
H2A3_12_17 S16ac	0.18%	0.03%	0.05%	0.03%	0.03%	0.02%	0.01%	0.03%	0.04%	0.00	0.00	0.00	0.00	0.00	0.00	0.00	0.00	0.00	0.45	1.00	0.15	0.196	1.000	0.431
H2A1_72_77 unmod	98.41%	98.76%	99.81%	96.93%	97.08%	99.88%	99.49%	99.87%	99.82%	0.01	0.01	0.00	0.05	0.03	0.00	0.00	0.00	0.00	0.61	0.34	0.31	0.431	0.196	0.516
H2A1_72_77 K74ac	1.59%	1.24%	0.19%	3.07%	2.92%	0.12%	0.51%	0.13%	0.18%	0.01	0.01	0.00	0.05	0.03	0.00	0.00	0.00	0.00	0.61	0.34	0.31	0.430	0.196	0.516
H2A_1_88 H2A14s.HLQLAIR	90.29%	93.22%	94.05%	93.24%	90.83%	86.91%	92.55%	89.10%	75.91%	0.04	0.01	0.02	0.00	0.04	0.04	0.02	0.02	0.12	0.36	0.43	0.05	0.575	0.555	0.199
H2A_1_88 H2AZ.AGGKAGKDSGKAKT	8.01%	5.69%	5.20%	5.85%	7.69%	11.35%	6.31%	3.25%	9.07%	0.04	0.01	0.02	0.01	0.03	0.04	0.02	0.02	0.08	0.48	0.39	0.05	0.735	0.108	0.658
H2A_1_88 H2AY.SAKAGVIFPVGR	0.20%	0.15%	0.24%	0.13%	0.20%	0.25%	0.17%	0.03%	0.00%	0.00	0.00	0.00	0.00	0.00	0.00	0.00	0.00	0.00	0.53	0.32	0.96	0.592	0.020	1.000
H2A_1_88 H2AX.GKTGGKAR	1.50%	0.94%	0.51%	0.78%	1.28%	1.49%	0.97%	7.61%	15.02%	0.00	0.01	0.00	0.00	0.01	0.00	0.00	0.03	0.04	0.03	0.59	0.01	0.540	0.035	0.031

Table S7. Effects of MGL50 on PTMs of H2B histone variant in HT-29 cells. Cells were seeded ($2.5x10^5$ cells/well) into 12-well plates, were treated with 0.03 mg/mL MGL (MGL50) for 24, 48 and 72 h and then were collected. Histones were extracted and peptides were analyzed by mass spectrometry. Data are represented as mean and standard deviation. NT, non-treated; PLP, control vehicle, cells treated with 0.5 mM PLP in PBS; MGL50, concentration of MGL able to reduce cell viability to 50%. Heteroscedastic two-tails t-test are applied and statistical analysis was carried out comparing, at each incubation time, NT vs PLP and PLP vs MGL. *= $p \leq 0.05$; **= $p \leq 0.01$ and ***= $p \leq 0.001$. Statistically significant differences are highlighted in green.

H2B variant	mean								
	NT 24h	NT 48h	NT 72h	PLP 24h	PLP 48h	PLP 72h	MGL50 24	MGL50 48	MGL50 72
H2B1B_80_86 unmod	99.94%	99.79%	99.88%	99.92%	99.73%	99.91%	99.67%	98.36%	96.15%
H2B1B_80_86 Y83ac	0.06%	0.21%	0.12%	0.08%	0.27%	0.09%	0.33%	1.64%	3.85%
H2B1A_81_87 unmod	6.86%	10.93%	21.22%	8.03%	5.86%	11.65%	20.11%	2.68%	19.54%
H2B1A_81_87 K86ac	93.14%	89.07%	78.78%	91.97%	94.14%	88.35%	79.89%	97.32%	80.46%
H2B_1_29 1C.PEPAKSAPAPKKGSKKAVTKAQK	44.39%	35.37%	56.40%	50.48%	57.04%	58.45%	54.73%	44.74%	48.22%
H2B_1_29 1H.PDPAKSAPAPKKGSKKAVTKAQ	9.41%	6.67%	11.96%	6.33%	5.18%	13.68%	9.05%	5.40%	5.32%
H2B_1_29 2F.PDPAKSAPAPKKGSKKAVTKVQ	2.22%	1.40%	5.59%	11.50%	0.80%	6.00%	3.32%	0.45%	10.38%
H2B_1_29 1B.PEPSKSAPAPKKGSKKAITKAQK	6.78%	32.65%	4.74%	6.79%	3.64%	0.02%	6.11%	32.62%	10.73%
H2B_1_29 1N.PEPSKSAPAPKKGSKKAVTKAQK	10.41%	8.69%	6.60%	4.27%	6.85%	5.23%	0.19%	0.00%	0.00%
H2B_1_29 1D.PEPTKSAPAPKKGSKKAVTKAQK	10.63%	6.95%	13.61%	7.81%	12.74%	14.25%	9.74%	4.85%	13.77%
H2B_1_29 1M.PEPVKSAPVPKKGSKKAINKAQ	3.80%	0.58%	1.10%	6.61%	10.70%	2.38%	16.64%	1.56%	11.58%
H2B_1_29 1L.PELAKSAPAPKKGSKKAVTKAQK	12.37%	7.68%	0.00%	6.21%	3.05%	0.00%	0.22%	10.36%	0.00%

H2B variant	standard deviation									NT vs PLP			PLP vs MGL		
	NT 24h	NT 48h	NT 72h	PLP 24h	PLP 48h	PLP 72h	MGL50 24	MGL50 48	MGL50 72	24h	48h	72h	24h	48h	72h
H2B1B_80_86 unmod	0.00	0.00	0.00	0.00	0.00	0.00	0.00	0.00	0.03	0.18	0.73	0.42	0.120	0.001	0.126
H2B1B_80_86 Y83ac	0.00	0.00	0.00	0.00	0.00	0.00	0.00	0.00	0.03	0.18	0.73	0.42	0.120	0.001	0.126
H2B1A_81_87 unmod	0.04	0.05	0.16	0.01	0.05	0.10	0.06	0.03	0.18	0.62	0.32	0.43	0.028	0.421	0.545
H2B1A_81_87 K86ac	0.04	0.05	0.16	0.01	0.05	0.10	0.06	0.03	0.18	0.62	0.32	0.43	0.028	0.421	0.545
H2B_1_29 1C.PEPAKSAPAPKKGSKKAVTKAQK	0.09	0.26	0.09	0.14	0.12	0.04	0.10	0.31	0.32	0.57	0.26	0.73	0.692	0.559	0.636
H2B_1_29 1H.PDPAKSAPAPKKGSKKAVTKAQ	0.03	0.06	0.03	0.06	0.08	0.02	0.12	0.09	0.09	0.47	0.81	0.45	0.751	0.976	0.201
H2B_1_29 2F.PDPAKSAPAPKKGSKKAVTKVQ	0.02	0.01	0.00	0.12	0.01	0.02	0.06	0.01	0.12	0.30	0.63	0.73	0.335	0.727	0.579
H2B_1_29 1B.PEPSKSAPAPKKGSKKAITKAQK	0.06	0.47	0.08	0.06	0.05	0.00	0.05	0.40	0.19	1.00	0.40	0.42	0.894	0.334	0.423
H2B_1_29 1N.PEPSKSAPAPKKGSKKAVTKAQK	0.07	0.08	0.11	0.06	0.06	0.09	0.00	0.00	0.00	0.30	0.76	0.88	0.360	1.000	1.000
H2B_1_29 1D.PEPTKSAPAPKKGSKKAVTKAQK	0.00	0.06	0.01	0.05	0.07	0.01	0.01	0.08	0.17	0.44	0.35	0.53	0.560	0.282	0.965
H2B_1_29 1M.PEPVKSAPVPKKGSKKAINKAQ	0.03	0.01	0.01	0.02	0.11	0.01	0.17	0.03	0.14	0.20	0.26	0.29	0.407	0.241	0.383
H2B_1_29 1L.PELAKSAPAPKKGSKKAVTKAQK	0.04	0.08	0.00	0.03	0.04	0.00	0.00	0.11	0.00	0.10	0.42	1.00	0.079	0.349	1.000

Table S8. Effects of MGL50 on PTMs of H3.3 histone variant in HT-29 cells. Cells were seeded (2.5×10^5 cells/well) into 12-well plates, were treated with 0.03 mg/mL MGL (MGL50) for 24, 48 and 72 h and then were collected. Histones were extracted and peptides were analyzed by mass spectrometry. Data are represented as mean and standard deviation. NT, non-treated; PLP, control vehicle, cells treated with 0.5 mM PLP in PBS; MGL50, concentration of MGL able to reduce cell viability to 50%. Heteroscedastic two-tails t-test are applied and statistical analysis was carried out comparing, at each incubation time, NT vs PLP and PLP vs MGL. *= p ≤ 0.05; **= p ≤ 0.01 and ***= p ≤ 0.001. Statistically significant differences are highlighted in green.

H3.3 variant	mean									standard deviation									NT vs PLP			PLP vs MGL		
	NT 24h	NT 48h	NT 72h	PLP 24h	PLP 48h	PLP 72h	MGL50 24	MGL50 48	MGL50 72	NT 24h	NT 48h	NT 72h	PLP 24h	PLP 48h	PLP 72h	MGL50 24	MGL50 48	MGL50 72	24h	48h	72h	24h	48h	72h
H33_27_40 unmod	3.41%	3.67%	2.89%	2.52%	3.26%	3.12%	10.14%	11.59%	7.42%	0.00	0.00	0.00	0.00	0.00	0.00	0.020	0.023	0.016	0.01	0.06	0.55	0.021	0.024	0.011
H33_27_40 K36me1	1.72%	2.34%	1.62%	1.45%	1.94%	1.59%	2.55%	1.34%	0.06%	0.00	0.00	0.00	0.00	0.00	0.00	0.004	0.004	0.001	0.18	0.03	0.92	0.007	0.089	0.000
H33_27_40 K27me1	2.95%	3.69%	2.64%	2.51%	3.19%	2.57%	4.47%	2.76%	0.52%	0.00	0.00	0.00	0.00	0.00	0.00	0.005	0.010	0.002	0.28	0.04	0.82	0.006	0.508	0.000
H33_27_40 K27me2	25.04%	14.79%	24.49%	23.52%	22.00%	26.37%	15.13%	25.68%	23.55%	0.01	0.01	0.01	0.01	0.03	0.01	0.016	0.080	0.058	0.14	0.02	0.09	0.001	0.498	0.451
H33_27_40 K36me2	23.46%	8.39%	21.82%	20.03%	9.00%	24.24%	8.34%	7.16%	2.57%	0.01	0.04	0.05	0.02	0.01	0.01	0.043	0.025	0.012	0.06	0.83	0.50	0.013	0.323	0.000
H33_27_40 K27me3	5.40%	6.03%	4.97%	4.81%	6.67%	5.10%	6.73%	7.31%	10.60%	0.00	0.00	0.01	0.00	0.01	0.00	0.008	0.012	0.021	0.05	0.20	0.85	0.017	0.435	0.043
H33_27_40 K36me3	0.10%	0.69%	0.06%	0.11%	0.10%	0.03%	0.08%	1.05%	0.81%	0.00	0.01	0.00	0.00	0.00	0.00	0.000	0.010	0.014	0.53	0.43	0.15	0.286	0.237	0.438
H33_27_40 K27me2K36me1	10.51%	13.40%	12.57%	12.74%	12.71%	12.08%	12.78%	10.87%	5.63%	0.01	0.01	0.01	0.00	0.00	0.02	0.010	0.022	0.045	0.01	0.27	0.74	0.950	0.238	0.089
H33_27_40 K27me1K36me2	5.30%	7.96%	6.56%	5.81%	6.95%	4.89%	7.24%	4.99%	1.05%	0.00	0.00	0.01	0.01	0.00	0.00	0.007	0.011	0.001	0.40	0.04	0.08	0.085	0.041	0.000
H33_27_40 K27me1K36me1	1.63%	3.38%	2.09%	2.09%	2.09%	1.56%	2.96%	0.13%	0.00%	0.01	0.01	0.00	0.00	0.01	0.00	0.007	0.001	0.000	0.53	0.06	0.19	0.098	0.005	1.000
H33_27_40 K27me3K36me1	2.23%	2.97%	1.95%	2.34%	2.84%	1.98%	3.25%	2.92%	1.43%	0.00	0.00	0.00	0.00	0.00	0.00	0.003	0.004	0.006	0.43	0.10	0.89	0.011	0.738	0.186
H33_27_40 K27me1K36me3	2.39%	3.85%	2.78%	2.35%	3.36%	2.19%	2.81%	1.70%	0.23%	0.00	0.00	0.01	0.00	0.00	0.00	0.002	0.004	0.002	0.72	0.01	0.22	0.040	0.002	0.000
H33_27_40 K27me2K36me2	10.64%	15.46%	10.77%	14.02%	15.32%	9.86%	15.03%	15.80%	17.02%	0.02	0.01	0.01	0.01	0.00	0.01	0.006	0.034	0.100	0.04	0.82	0.23	0.193	0.833	0.340
H33_27_40 K27me3K36me2	3.09%	4.49%	2.68%	3.71%	4.31%	2.58%	3.92%	3.47%	2.26%	0.00	0.00	0.00	0.00	0.00	0.00	0.002	0.007	0.016	0.13	0.42	0.77	0.392	0.118	0.749
H33_27_40 K27ac	0.53%	0.52%	0.50%	0.42%	0.50%	0.40%	0.81%	0.86%	0.75%	0.00	0.00	0.00	0.00	0.00	0.00	0.001	0.002	0.006	0.12	0.67	0.31	0.003	0.044	0.451
H33_27_40 S28ph	0.07%	0.82%	0.40%	0.35%	0.98%	0.06%	0.72%	1.28%	25.77%	0.00	0.00	0.00	0.00	0.00	0.00	0.002	0.009	0.223	0.02	0.53	0.00	0.072	0.633	0.184
H33_27_40 K27me1S28ph	0.00%	0.00%	0.00%	0.00%	0.00%	0.00%	0.00%	0.00%	0.00%	0.00	0.00	0.00	0.00	0.00	0.00	0.000	0.000	0.000	1.00	1.00	1.00	1.000	1.000	1.000
H33_27_40 K27me2S28ph	0.09%	0.01%	0.00%	0.05%	0.05%	0.03%	0.00%	0.00%	0.00%	0.00	0.00	0.00	0.00	0.00	0.00	0.000	0.000	0.000	0.43	0.19	1.00	0.265	1.000	1.000
H33_27_40 K27me3S28ph	0.04%	0.01%	0.01%	0.05%	0.10%	0.02%	0.02%	0.00%	0.00%	0.00	0.00	0.00	0.00	0.00	0.00	0.000	0.000	0.000	0.53	0.29	0.12	0.100	0.281	1.000
H33_27_40 K27me2S28phK36me	0.04%	0.00%	0.01%	0.05%	0.01%	0.01%	0.01%	0.00%	0.00%	0.00	0.00	0.00	0.00	0.00	0.00	0.000	0.000	0.000	0.71	0.56	0.52	0.050	1.000	1.000
H33_27_40 K27me3S28phK36me	0.00%	0.13%	0.00%	0.00%	0.15%	0.00%	0.00%	0.00%	0.00%	0.00	0.00	0.00	0.00	0.00	0.00	0.000	0.000	0.000	0.52	0.89	1.00	1.000	1.000	1.000
H33_27_40 S28ac	0.47%	5.90%	0.20%	0.17%	3.33%	0.02%	1.85%	0.42%	0.00%	0.01	0.03	0.00	0.00	0.02	0.00	0.007	0.007	0.000	0.54	0.26	0.31	0.015	0.051	1.000
H33_27_40 K27me2S28ac	0.16%	0.02%	0.08%	0.20%	0.12%	0.51%	0.04%	0.02%	0.00%	0.00	0.00	0.00	0.00	0.00	0.00	0.000	0.000	0.000	0.64	0.23	0.16	0.131	0.207	1.000
H33_27_40 K27me3S28ac	0.42%	0.84%	0.55%	0.39%	0.62%	0.50%	0.63%	0.54%	0.34%	0.00	0.00	0.00	0.00	0.00	0.00	0.002	0.001	0.004	0.84	0.25	0.55	0.097	0.434	0.497
H33_27_40 K27me3S28acK36me	0.31%	0.65%	0.37%	0.29%	0.39%	0.28%	0.48%	0.10%	0.00%	0.00	0.00	0.00	0.00	0.00	0.00	0.001	0.001	0.000	0.83	0.09	0.17	0.053	0.011	1.000
